# An Account of a Large Mass of Hydatids Discharged from the Uterus

**Published:** 1787

**Authors:** B. Wilmer

**Affiliations:** Surgeon at Coventry


III. *An Account of a large Mass of Hydatids discharged from the Uterus. Communicated in a Letter to Dr.* Simmons *by Mr.* B. Wilmer, *Surgeon at Coventry.*

MRS. Oakes, of Longford, in the county of Warwick, aged forty-six years, had a healthy child born about two years since, and from that period, till the month of April last, she

ſhe continued in good health. I was then deſired to viſit her. She informed me that ſhe had reaſon to believe ſhe was between four and five months gone with child; her menſes, till lately, had been ſuppreſſed; her breaſts were turgid, and diſcharged milk from the nipples; and there was an enlargement of the lower part of the abdomen. During the laſt fourteen days ſhe had, at intervals, floodings, which, now recurring more frequently, had reduced her ſtrength much. Her countenance was pale and bloated, and a troubleſome cough allowed her to get no ſleep in the night.

I enjoined reſt, directed a draught of tincture of roſes, to which ten drops of laudanum were added, to be taken every ſix hours; and, at bed-time, ſhe took a medicine compoſed of a ſolution of ſpermaceti with a ſmall quantity of paregoric elixir.

On the following day I was haſtily ſent for, the meſſenger informing me that, unleſs I was expeditious, I ſhould not find my patient alive. She had been ſeized with addition of pain and flooding, and, before I arrived, had been delivered of what her attendants called a falſe conception. It nearly filled a common-ſized chamber ber pot, and, upon examination, I perceived it was a mafs of hydatids, of various fizes, but none of them were larger than a horfe bean: they were perfectly round, and clufterd together like a bunch of grapes, with this difference, that they did not adhere by a common peduncle, but the whole mafs, wherever it was broke or cut through, appeared to be compofed of them.

Mrs. Oakes was in bed, and fo much reduced by the difcharge fhe had fuffered, and the fatigue fhe had undergone, joined to her general bad ftate of health, that I was extremely apprehenfive for the confequences. The flooding, though diminifhed, ftill continued; pains of the abdomen, at times, returned; and on the following, as well as during feveral fucceeding days, fmall bunches of hydatids were difcharged from the uterus.

By means of a nourifhing diet, the bark, cool air, &c., fhe began to recover her ftrength. At the expiration of a week no more hydatids were feen, and the difcharge had nearly ceafed. In a few weeks fhe was able to walk; her cough left her, and fhe is now as well as before her confinement.

Some

Some years ago I visited a patient, with Mr. Cole, Surgeon, of this city, whose case was nearly similar to the above.

This case is described by Ruysch *, who supposes the hydatids are produced by a diseased state of the glands of a retained placenta; but in the above instances the mass discharged appeared to consist entirely of hydatids, connected by a mucous medium.

*Coventry,*

Sept. 27th, 1787.

